# Editorial: Etiology and treatment for children and adolescents with autism spectrum disorder

**DOI:** 10.3389/fpsyt.2023.1222384

**Published:** 2023-07-13

**Authors:** Fei-Yong Jia, Ting-Yu Li

**Affiliations:** ^1^Department of Developmental and Behavioral Pediatrics, The First Hospital of Jilin University, Jilin University, Changchun, China; ^2^Chongqing Key Laboratory of Child Nutrition and Health, Ministry of Education Key Laboratory of Child Development and Disorders, National Clinical Research Center of Child Health and Disorders, Children's Hospital of Chongqing Medical University, Chongqing, China

**Keywords:** autism spectrum disorder, etiology, treatment, children, intervention

## Introduction

Autism spectrum disorder (ASD) is a neurodevelopmental disorder characterized by social communication impairments and restricted, repetitive behavior (1). However, the etiology of ASD is under investigation and the treatment is still challenging. The current topic, therefore, aims to focus on the etiology and treatment of children and adolescents with ASD.

## Etiology for ASD

The etiology of ASD is not incompletely understood, which can be interpreted as gene-environment interaction contributing to autism risk (2). The general consensus is that ASD is a collection of related disorders of different etiologies (1). Maternal and early developmental vitamin D (VD) may be associated with ASD (3). Muskens et al. found 75.9% of the children with ASD had VD deficiency. But The mechanism of VD in ASD is still unclear. Wang X. et al. reviewed current studies and found various factors (including abnormalities in neurotransmitters, immune disorders, etc.) may cause abnormal levels of nitric oxide (NO) during the critical period of brain development which was an important factor in the pathogenesis of ASD. VD can regulate the levels of molecules in the NO signaling pathway. Wang B. et al. hypothesized a potential mechanism that VD affects the pathogenesis and severity of ASD by regulating NO levels. Thus, monitoring maternal and early developmental NO levels may become an important strategy for ASD risk prediction in clinical practice in the future. Pichugina et al. compared salivary oxytocin (OT) concentration between ASD children with intellectual disability (ID) and children with ID. The results indicated that children with ID and ASD demonstrated a lower level of salivary OT concentration and a direct relationship between low salivary OT level and a high degree of severity of ASD. Research on the effect of OT on ASD with ID was limited. Further studies on the relationship between OT and ASD with ID are warranted. Chen et al. found children with ASD had an altered microbial community structure of lower alpha diversity indices and higher Bacteroides and Faecalibacterium than children with ID or typically developing. The gut microbiota may modulate central nervous system activities through neural, immune, and endocrine pathways thereby affecting ASD. Further research is needed on the relationship between gut microbiota and the etiology of ASD. Qin et al. investigated the resting-state functional brain network characteristics of preschool children with ASD and found evident alterations in functional brain networks. Functional connectivity changed mainly in the default mode network (DMN), hippocampus, and parahippocampal gyrus. The study provided some evidence on understanding the brain functional pathogenesis of ASD.

## Treatment for ASD

Children with ASD generally require comprehensive treatments including nonpharmacological and pharmacological treatments (4). The current evidence-based management of ASD in children relies primarily on behavioral treatments to modify the core symptoms of ASD. Parent-mediated intervention, delivered by trained parents is a potential treatment (5). Li et al. evaluate the effectiveness of an 8-week, online-delivered project Improve Parents as Communication Teacher (ImPACT) program for children with ASD and their parents in China during the COVID-19 pandemic. They have found social communication skills of children with ASD significantly improved and the parenting stress of parents decreased. This study provided evidence that parents' training delivered online may be a promising program to promote intervention accessibility when face-to-face training is unavailable. Gao et al. used Repetitive transcranial magnetic stimulation (rTMS) on the bilateral dorsolateral prefrontal cortex (DLPFC) of children with ASD and found the core symptoms and sleep problems improved significantly after two courses of intervention of rTMS. They also found that sensory abnormality mediated the improvement of rTMS on sleep problems of ASD. Current measures for managing sleep disorders in ASD are sleep education, sleep environment preparation, behavioral intervention, and drug treatment (such as melatonin) (6). The study provided new ideas for the intervention of sleep problems in ASD. Leuning et al. gave 21 adolescents with ASD a ten-session Eye Movement Desensitization and Reprocessing (EMDR) treatment on daily experienced stress and found EMDR treatment significantly reduced perceived stress as reported by the participants, and improves global clinical functioning. This study provided novel ideas for the treatment of core symptoms and experienced stress in adolescents with ASD. The stem cell is receiving attention as a potential therapy for ASD, but the efficacy and safety of stem cell is unclear (7). Qu et al. performed a meta-analysis of stem cell therapy for children with ASD and found that stem cell therapy might be safe and effective. However, the evidence was insufficient due to many factors, such as the small size of studies, varied injection routes, and doses of stem cells. There are still many problems with stem cell therapy for ASD, and a lot of work needed to be done.

## Conclusions

The current work explores the etiology and treatment of ASD. These results support that ASD is a neurodevelopmental disorder with complex components. Vitamin D, OT, gut microbiota, and functional brain network may influence ASD through different pathways. Further research is still needed on the pathogenesis of ASD. Parent-mediated intervention is still important in clinical practice. There are various forms of parental training, and online teaching is a good choice in clinical practice. Other nonpharmacological treatments can serve as a supplement to behavioral interventions to improve core symptoms and comorbid problems of ASD.

## Author contributions

All authors listed have made a substantial, direct, and intellectual contribution to the work and approved it for publication.

## References

[1] Hirota T King BH. Autism spectrum disorder: a review. JAMA. (2023) 329:157–168. 10.1001/jama.2022.23661. 36625807

[2] Bai D Yip BHK Windham GC Sourander A Francis R Yoffe R. Association of genetic and environmental factors with autism in a 5-Country Cohort. JAMA Psychiatry. (2019) 76:1035–43. 10.1001/jamapsychiatry.2019.1411. 31314057 PMC6646998

[3] Principi N Esposito S. Vitamin D deficiency during pregnancy and autism spectrum disorders development. Front Psychiatry. (2020) 10:987. 10.3389/fpsyt.2019.00987. 32082196 PMC7006052

[4] Aishworiya R Valica T Hagerman R Restrepo B. An update on psychopharmacological treatment of autism spectrum disorder. Neurotherapeutics. (2022) 19:248–62. 10.1007/s13311-022-01183-1. 35029811 PMC9130393

[5] Brian J Solish A Dowds E Roth I Bernardi K Perry K. Effectiveness of a parent-mediated intervention for toddlers with autism spectrum disorder: evidence from a large community implementation. Autism. (2022) 26:1882–97. 10.1177/13623613211068934. 35037520

[6] Schwichtenberg AJ Janis A Lindsay A Desai H Sahu A Kellerman A. Sleep in children with autism spectrum disorder: a narrative review and systematic update. Curr Sleep Med Rep. (2022) 8:51–61. 10.1007/s40675-022-00234-5. 36345553 PMC9630805

[7] Paprocka J Kaminiów K Kozak S Sztuba K Emich-Widera E. Stem cell therapies for cerebral palsy and autism spectrum disorder-a systematic review. Brain Sci. (2021) 11:1606. 10.3390/brainsci11121606. 34942908 PMC8699362

